# HIV-1 transmitted drug resistance in Slovenia and its impact on predicted treatment effectiveness: 2011–2016 update

**DOI:** 10.1371/journal.pone.0196670

**Published:** 2018-04-26

**Authors:** Maja M. Lunar, Snježana Židovec Lepej, Janez Tomažič, Tomaž D. Vovko, Blaž Pečavar, Gabriele Turel, Manja Maver, Mario Poljak

**Affiliations:** 1 Institute of Microbiology and Immunology, Faculty of Medicine, University of Ljubljana, Ljubljana, Slovenia; 2 Dr. Fran Mihaljević University Hospital for Infectious Diseases, Zagreb, Croatia; 3 Department of Infectious Diseases, Ljubljana University Medical Center, Ljubljana, Slovenia; Institut Pasteur of Shanghai Chinese Academy of Sciences, CHINA

## Abstract

HIV-positive individuals that have a detected transmitted drug resistance (TDR) at baseline have a higher risk of virological failure with antiretroviral therapy (ART). This study offers an update on the prevalence of TDR in Slovenia, looks for onward transmission of TDR, and reassesses the need for baseline drug resistance testing. Blinded questionnaires and partial *pol* sequences were obtained from 54.5% (168/308) of all of the patients diagnosed with HIV-1 from 2011 to 2016. Subtype B was detected in 82.7% (139/168) of patients, followed by subtype A (8.3%), subtype C (2.4%), and CRF01_AE (1.8%). Surveillance drug resistance mutations (SDRMs) were found in four individuals (2.4%), all of them men who have sex with men (MSM) and infected with subtype B. K103N was detected in two patients and T68D and T215D in one person each, corresponding to a prevalence of 0%, 1.2%, and 1.2% of TDR to protease inhibitors (PIs), nucleoside reverse transcriptase inhibitors (NRTIs), and non-NRTIs (NNRTIs), respectively. The impact of mutations on drug susceptibility was found to be most pronounced for NNRTIs. No forward spread of TDR within the country was observed; however, phylogenetic analysis revealed several new introductions of HIV into Slovenia in recent years, possibly due to increased risky behavior by MSM. This was indirectly confirmed by a substantial increase in syphilis cases and HIV-1 non-B subtypes during the study period. A drug-resistant HIV variant with good transmission fitness is thus more likely to be imported into Slovenia in the near future, and so TDR should be closely monitored.

## Introduction

Great efforts to provide HIV antiretroviral therapy (ART) to all people living with HIV (PLWH) have been made globally. The World Health Organization (WHO) has promoted this goal under the target of 90-90-90; that is, 90% of PLWH diagnosed, 90% of diagnosed PLWH receiving ART, and 90% of them achieving viral suppression by 2020. By the end of 2016, the WHO estimated that this goal is yet to be reached, with the current proportions at 70-77-82 [1]. Even though identifying all PLWH and providing them with ART is of outmost importance, this is extremely challenging. On the other hand, a significant impact on the third “90” (i.e., ART effectiveness) can be made by promoting good adherence and thus reducing the risk of emergence of acquired drug resistance coupled with active national and regional monitoring and baseline (pre-treatment) testing of transmitted drug resistance (TDR). A higher risk of virological failure has been observed among PLWH with detected TDR even when only low-level resistance was present at baseline [2].

A rising trend of TDR in low- and middle-income countries (LMIC) has been observed because ART has only recently started becoming available there. The prevalence of TDR in LMIC nearly doubled from 2009 to 2013 in comparison to 2004 to 2008. In contrast, a stabilizing trend has been noted in high-income countries [3]. Hofstra et al. (2016) presented data from an international study on monitoring TDR in Europe (the SPREAD program) and found an overall prevalence of 8.3% from 2008 to 2010, confirming the previous observation of TDR stabilizing across Europe. The study concluded that most detected mutations conferred resistance to nucleoside reverse transcriptase inhibitors (NRTIs); however, the predicted impact of TDR mutations was greatest regarding susceptibility to non-nucleoside reverse transcriptase inhibitor (NNRTI)–based regimens. The NRTI mutations did not affect the predicted susceptibility of the currently used regimen; however, susceptibility was reduced to inhibitors added to the NRTI backbone—specifically, by mutations conferring resistance to NNRTIs or protease inhibitors (PIs) [4].

High levels of TDR can result from onward transmission of strains carrying TDR mutations within a country, as was shown in a recent study by Paraskevis et al. (2017). A high prevalence of NNRTI resistance was found in HIV-1 subtype A as a result of local Greek transmission networks [5]. A similar finding was observed in Croatia, which borders Slovenia, where a TDR prevalence of an alarming 22% was assessed, primarily due to the onward transmission of T215S revertant mutation [6]. Data from other neighboring countries and regions also showed a higher prevalence of TDR among treatment-naive individuals than the reported European average; specifically, 17% and 12% in Hungary and northern Italy, respectively [7,8].

In contrast, Slovenia, which is a small central European country with a population of 2 million, had a historically low TDR prevalence (i.e., 4.7% TDR among HIV-1 positive individuals diagnosed from 2005 to 2010), which is similar to the rates reported from some Balkan countries; for example, from Bulgaria (5.2%), Serbia (8.8%), and Greece (6.0%) [5,9–11].

This study offers an update on the national prevalence of TDR in the last 6 years in Slovenia (2011–2016), examines whether cases of TDR are due to onward transmission of strains carrying TDR mutations within the country, and reassesses the need for baseline drug resistance testing before ART initiation. In addition, it assesses the impact of baseline drug resistance mutations on the treatment effectiveness of currently recommended regimens.

## Materials and methods

### Prevalence of TDR

Altogether, 308 patients were diagnosed with HIV-1 between January 2011 and December 2016 in Slovenia. Blinded questionnaires were approved by the Medical Ethics Committee at the Ministry of Health of Slovenia (ref. no. 126/12/03) and were collected for 238 individuals. Slovenian legislation does not require informed consent in this case because the research was not carried out on human subjects and the data were anonymized prior the analysis. Afterwards, 168 (54.5%) plasma samples were randomly selected, but within the sex and mode of transmission stratum, and subjected to population-based Sanger sequencing as previously reported [9]. TDR was assessed annually, after completion of each calendar year, and analyzed according to the WHO’s drug-resistance mutations for surveillance of the TDR 2009 update. Drug-resistant mutations indicating TDR were termed surveillance drug-resistance mutations (SDRMs) on the WHO list to unify TDR reporting between programs and countries [12].

The HIV-1 subtype was determined by using Rega version 3.0, Comet version 2.2, jpHMM-HIV, and SCUEAL [13–16].

Fisher’s exact test was used for categorical data and *t*-statistics for continuous data using OpenEpi version 3.01; associations with *p* < 0.05 were considered significant [17].

### Genotypic sensitivity score

The genotypic sensitivity score (GSS) was calculated for the 168 sequences of the 2011–2016 dataset, as previously described [4]. Briefly, susceptibility assessment as provided by the Stanford HIVdb Program version 8.4 was scored 0, 0.5, and 1 for high-level resistance, intermediate or low-level resistance, and potential low-level resistance or susceptibility to each relevant drug, respectively [4,18]. To obtain a final score for the first-line drug combinations currently recommended in Slovenia, all of the GSSs of the individual drugs included in the regimen were added [4]. Integrase strand transfer inhibitors (INSTIs) were not included in the analysis because integrase was not sequenced for the majority of HIV-positive patients included in the study. However, in order to calculate the GSSs of the regimen including INSTIs, the GSSs of each INSTI were presumed to be one. However, 46 baseline sequences of the integrase region were available from 2000 to 2016 and were inspected for predicted INSTI effectiveness.

In addition, the GSS was calculated for 238 sequences obtained from treatment-naive individuals diagnosed from 2000 to 2010 to monitor whether changes in treatment effectiveness have occurred in recent years. The use of these data was approved by the Medical Ethics Committee at the Slovenian Ministry of Health (ref. no. 126/12/03). This dataset was primarily comprised of men (87.8%) diagnosed at a mean age of 38.2 ± 11.9 years. Subtype B was determined as the most prevalent subtype (84.5%), followed by subtype A (5.5%) and CRF02_AG (2.1%). Most of these sequences were included in two previously published studies of TDR in Slovenia examining the periods from 2000 to 2004 and from 2005 to 2010 [9,19].

### Phylogenetic analyses

Sequences subtyped B/B-like and A were selected for separate phylogenetic inferences. Analysis for subtype B included 139 sequences obtained from individuals diagnosed with HIV-1 from 2011 to 2016 and 188 sequences from individuals diagnosed from 2000 to 2010 in Slovenia along with four reference sequences of subtype A to root the phylogenetic tree. All of the Slovenian sequences were subjected to BLAST search, and the 10 closest sequences were selected for background control sequences [20]. After removing duplicate sequences, the phylogenetic tree was inferred from a total of 774 sequences with a length of 987 base pairs using PhyML 3.0 with an integrated model selection following the Akaike Information Criterion (AIC) [21].

The subtype A phylogenetic tree was constructed with 14 sequences from this analysis, an additional 11 sequences from individuals diagnosed with HIV-1 from 2000 to 2010, and four subtype B reference sequences to root the tree. Once again, the BLAST tool was used to select for background sequences, and thus finally a total of 150 sequences were included in the analysis, executed as for subtype B, described above.

The phylogenetic cluster was defined according to approximate likelihood ratio test value (aLRT) > 0.95. Only clusters with at least three Slovenian sequences were considered to constitute an ongoing transmission within the country. Trees were visualized using Figtree version 1.4.3 (http://tree.bio.ed.ac.uk/software/figtree/).

Phylogenetic analyses were repeated after removing 43 SDRM sites (PR: 23, 24, 30, 32, 46, 47, 48, 50, 53, 54, 73, 76, 82, 83, 84, 85, 88, 90; RT: 41, 65, 67, 69, 70, 74, 75, 77, 100, 101, 103, 106, 115, 116, 151, 179, 181, 184, 188, 190, 210, 215, 219, 225, 230) on a final length of 858 base pairs in order to eliminate biased results due to drug resistance.

## Results

### Characteristics of the 2011–2016 dataset

The dataset analyzed contained a total of 168 patients: 150 men (89.3%), 17 women (10.1%), and one transgender person (0.6%). Individuals were diagnosed with HIV-1 at a mean age of 38.5 years with an average HIV-1 plasma viremia of 4.9 log copies/ml and a CD4 cell count of 326 cells/mm³ (Table 1).

**Table 1:** Characteristics of individuals diagnosed with HIV-1 in Slovenia, 2011–2016.

	Total population	%	Subtype B	%	Non-B	%	*p*-value
Subjects	168		139	83%	29	17%	
Sex							
Male	150	89%	132	95%	18	62%	**< 0.0001**
Female	17	10%	6	4%	11	38%	**< 0.0001**
Transgender	1	0.6%	1	1%	0	0%	> 0.9999
Age at diagnosis (mean, years ± SD)	38.5 ± 11.3		38.4 ± 11.4		39.0 ± 10.8		0.7889
Nationality							
Slovenia	152	90%	130	94%	22	76%	**0.0169**
Other	16	10%	9	6%	7	24%	
HIV test performed in the past							
Yes	83	49%	73	53%	10	34%	0.1169
No	65	39%	50	36%	15	52%	0.1712
Unknown	20	12%	16	12%	4	14%	
Acute retroviral syndrome							
Yes	35	21%	32	23%	3	10%	0.1919
No	101	60%	83	60%	18	62%	0.9857
Unknown	32	19%	24	17%	8	28%	
CDC class							
A	117	70%	98	71%	19	66%	0.7444
B	12	7.1%	11	7.9%	1	3.4%	0.7021
C	39	23%	30	22%	9	31%	0.3883
AIDS-defining illnesses							
Yes	36	21%	27	19%	9	31%	0.2580
No	132	79%	112	81%	20	69%	
Other sexually transmitted disease							
Yes	51	30%	46	33%	5	17%	0.1347
No	113	67%	90	65%	23	79%	0.1878
Unknown	4	2.4%	3	2.2%	1	3.4%	
Type of STD							
*Chlamydia trachomatis*	1	0.6%	1	0.7%	0	0%	> 0.9999
Genital and perianal warts	3	1.8%	3	2.2%	0	0%	> 0.9999
Gonorrhea	9	5.4%	7	5.0%	2	6.9%	0.9619
Genital or anal herpes	3	1.8%	3	2.2%	0	0%	> 0.9999
Syphilis	35	21%	34	24%	1	3.4%	**0.0117**
Coinfection							
Hepatitis B	41	24%	34	24%	7	24%	> 0.9999
Hepatitis C	7	4.2%	5	3.6%	2	6.9%	0.7224
Route of HIV infection							
Homosexual/bisexual contact	129	77%	121	87%	8	28%	**< 0.0001**
Heterosexual contact	34	20%	15	11%	19	66%	**< 0.0001**
IDU	2	1.2%	1	0.7%	1	3.4%	0.6326
Other/unknown	3	1.8%	2	1.4%	1	3.4%	0.8510
Relationship with source							
Sex with anonymous person	97	58%	85	61%	12	41%	0.0807
Stable relationship with source	39	23%	25	18%	14	48%	**0.0019**
Sex for money/drugs	4	2.4%	4	2.9%	0	0%	0.9302
Unknown / not applicable	35	21%	30	22%	5	17%	
Origin of the infection							
Slovenia	114	68%	98	71%	16	55%	0.1684
Other	36	21%	26	19%	10	34%	0.1104
Unknown	18	11%	15	11%	3	10%	
Viral load (mean, log ± SD)	4.9 ± 1.0		5.0 ± 1.0		4.7 ± 1.0		0.1493
CD4+ (mean, cells/mm³ ± SD)	326 ± 236		332 ± 234		302 ± 247		0.5518
< 200 cells/mm³	58	35%	47	34%	11	38%	0.8234
≥ 200 cells/mm³	110	65%	92	66%	18	62%	
SDRMs found	4	2.4%	4	2.9%	0	0%	0.9302

CDC = Center for Disease Control and Prevention; IDU = injecting drug use; SD = standard deviation; SDRMs = surveillance drug resistance mutations; STD = sexually transmitted disease.

Subtype B was present in 82.7% (139/168), subtype A in 8.3% (14/168), subtype C in 2.4% (4/168), CRF01_AE in 1.8% (3/168), and subtype F1 and CRF02_AG in one person each (0.6%). It was not possible to assign a subtype to the remaining six sequences (3.6%), which indicates potential novel recombinant forms. Infection with subtype B, as opposed to non-B subtype, was significantly associated with male sex, Slovenian nationality, syphilis coinfection, men who have sex with men (MSM) as a risk behavior, and not being in a stable relationship with the presumed HIV source (*p* < 0.0001, *p* = 0.0169, *p* = 0.0117, *p* < 0.0001, and *p* = 0.0019, respectively). Among the 35 patients with syphilis, 33 (94%) reported to be MSM and two reported a heterosexual mode of HIV acquisition (one male and one female; Table 1).

On the other hand, non-B subtypes were determined in a higher proportion of individuals of foreign origin and females, with heterosexual contact as the predominant risk factor (*p* = 0.0169, *p* < 0.0001, and *p* < 0.0001, respectively). In addition, a stable relationship with the source was reported significantly more often among non-B infected patients (*p* = 0.0019; Table 1).

### Prevalence of TDR

SDRMs were only found in four individuals (2.4%); namely, mutation K103N in two patients and T68D and T215D in one each. Thus, a prevalence of 0%, 1.2%, and 1.2% of TDR was determined for the PI, NRTI, and NNRTI drug classes, respectively (Table 2). All sequences with SDRMs were obtained from MSM infected with HIV-1 subtype B; however, these variables were not significantly associated with the detection of SDRMs (*p* = 0.6877 and *p* = 0.9302, respectively). Both individuals with NRTI TDR were diagnosed with an advanced stage of HIV (stage C). Three out of four patients presumably acquired HIV in Slovenia, and one person most probably acquired it in Brazil (Table 2).

**Table 2:** Characteristics of individuals diagnosed with HIV-1 from 2011 to 2016 that had surveillance drug resistance mutations (SDRMs) detected at baseline.

Year of diagnosis	Nationality	Country of infection	Transmission risk	HIV subtype	SDRM drug class	SDRM
2011	Slovenian	Slovenia	MSM	B	NRTI	T69D
2011	Slovenian	Slovenia	MSM	B	NRTI	T215D
2011	Slovenian	Brazil	MSM	B	NNRTI	K103N
2016	Slovenian	Slovenia	MSM	B	NNRTI	K103N

MSM = men who have sex with men; NRTI = nucleoside reverse transcriptase inhibitor; NNRTI = non-nucleoside reverse transcriptase inhibitor; SDRM = surveillance drug resistance mutation.

### Genotypic sensitivity score

**Fig 1:**
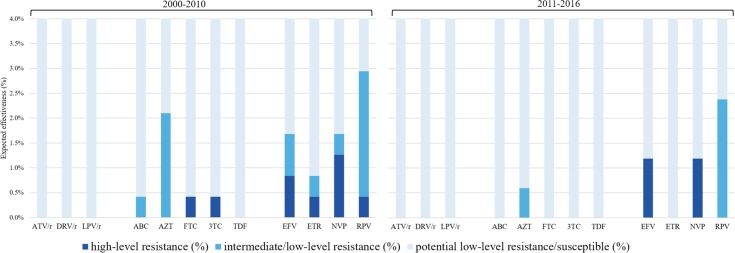
Genotypic sensitivity scores (GSSs) of individual antiretroviral drugs as estimated from sequences obtained from individuals diagnosed with HIV-1 in Slovenia, 2000–2010 and 2011–2016. ATV = atazanavir; r = ritonavir (protease inhibitor booster); DRV = darunavir; LPV = lopinavir; ABC = abacavir; AZT = zidovudine; FTC = emtricitabine; 3TC = lamivudine; TDF = tenofovir; EFV = efavirenz; ETR = etravirine; NVP = nevirapine; RPV = rilpivirine.

The GSSs of individual drugs for 2000–2010 and 2011–2016 are presented in Fig 1. All high-level resistance among patients diagnosed from 2011 to 2016 was predicted for the NNRTI drug class only. Among 387 patients diagnosed from 2000 to 2016, predicted high-level resistance was observed in 1.2% and 1.0% to nevirapine and efavirenz, respectively. High-level resistance to emtricitabine, lamivudine, etravirine, and rilpivirine was detected in a one individual (0.3%) only. The majority of intermediate and low-level resistance was detected to rilpivirine (2.5%; Fig 1). All 46 available baseline sequences of the integrase region scored 1 for all of the INSTIs available, suggesting no reduction in susceptibility.

The combined GSSs of drug combinations currently recommended as first-line treatment in Slovenia predicted success of treatment in ≥ 97% of individuals diagnosed from 2000 to 2016 (Table 3). Tenofovir/emtricitabine/rilpivirine (TDF/FTC/RPV) exhibited a GSS of 2.5 in 2.5% of patients due to low-level resistance to rilpivirine, and a GSS of 2.0 was predicted for two individuals (0.5%). A GSS of 1.5 was observed in one person (0.2%) only, specifically for a combination of abacavir/lamivudine/integrase strand inhibitor (ABC/3TC/INSTI). The GSSs from sequences obtained from 2011 to 2016 showed predicted 100% success of treatment for all combinations, except for TDF/FTC/RPV, due to low-level resistance to rilpivirine. On the other hand, among those diagnosed from 2000 to 2010, GSSs of 2.0 and 1.5 were observed among all recommended regimens (Fig 1).

**Table 3:** Distribution of genotypic sensitivity scores of recommended first-line regimen as predicted from baseline HIV protease and reverse-transcriptase sequences obtained from individuals diagnosed with HIV-1 in Slovenia, 2000 to 2016.

	Genotypic sensitivity scores: GSS (%)			
	3	2.5	2	1.5
**2 NRTI+INSTI**				
ABC+3TC+INSTI	99.8	0	0	0.2
TDF+FTC+INSTI	99.8	0	0.2	0
**2 NRTI+NNRTI**				
TDF+FTC+RPV	97.0	2.5	0.5	0
**2 NRTI+PI/r**				
TDF+FTC+DRV/r	99.8	0	0.2	0

3TC = lamivudine; ABC = abacavir; DRV = darunavir; FTC = emtricitabine; GSS = genotypic sensitivity score; INSTI = integrase strand transfer inhibitor; NRTI = nucleoside reverse transcriptase inhibitor; NNRTI = non-nucleoside reverse transcriptase inhibitor; PI = protease inhibitor; r = ritonavir (PI booster); RPV = rilpivirine; TDF = tenofovir.

### Phylogenetic analyses

**Fig 2:**
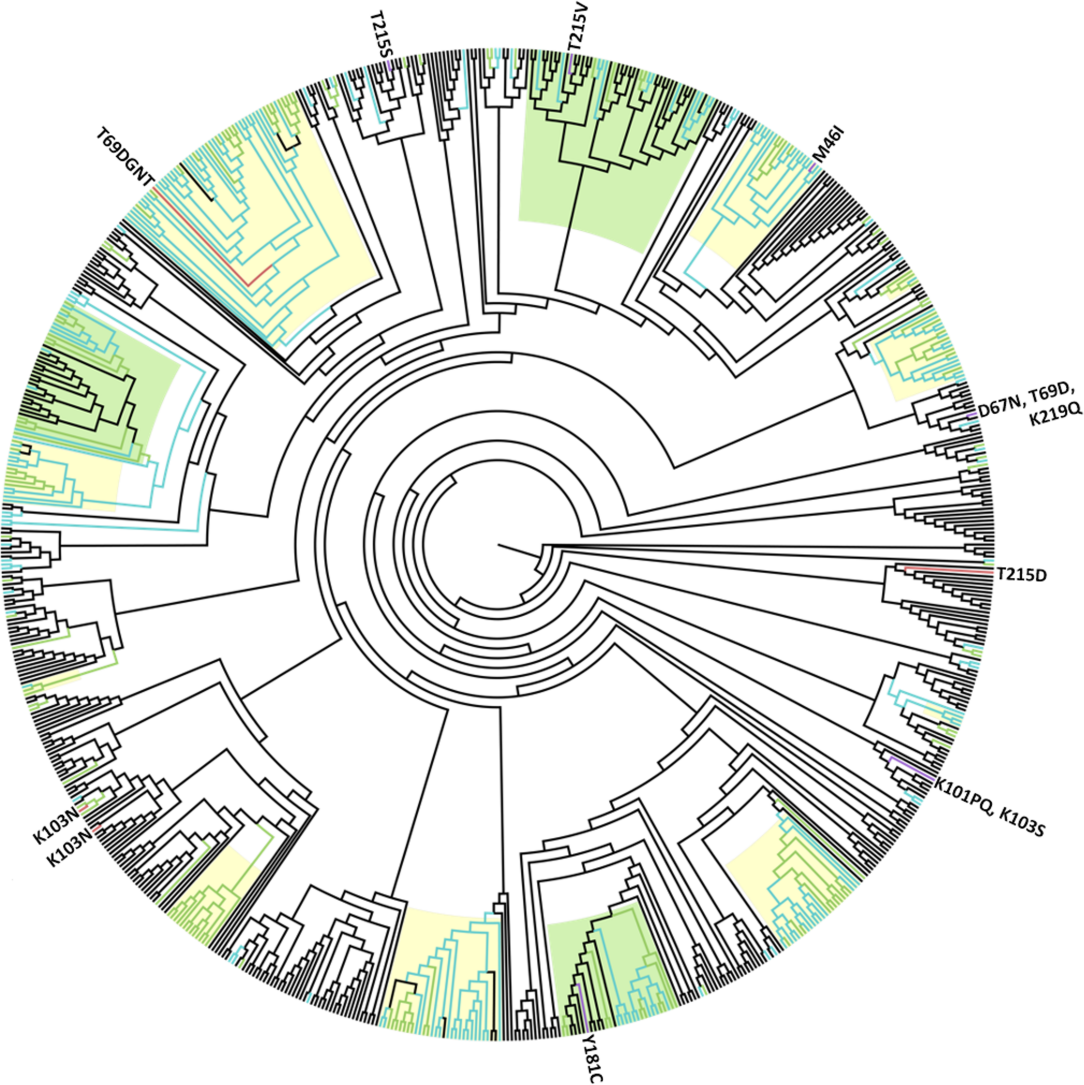
Maximum likelihood phylogenetic tree of Slovenian sequences subtyped B or B-like and corresponding control sequences. Slovenian sequences from the 2011–2016 dataset are colored green, with sequences carrying surveillance drug resistance mutations (SDRMs) colored red; sequences from the 2000–2010 dataset are colored blue, with sequences carrying SDRMs colored purple; and control sequences are shown in black. Identified SDRMs are depicted next to the corresponding sequences. Phylogenetic clusters with approximate likelihood ratio test values (aLRT) > 0.95 are highlighted; clusters with ≥ 90% of Slovenian sequences are highlighted yellow and clusters with > 10% of foreign sequences are highlighted green.

A phylogenetic tree of subtype B sequences was constructed using a generalized time-reversible model with gamma distribution of the rate variation and a significant proportion of invariable sites (model GTR+G+I) selected as the best-fitted model using the AIC criterion implemented in the PhyML 3.0 (Fig 2). Sequences from this analysis and those from the 2000–2010 dataset were colored differently in the phylogenetic tree in order to show which clusters have been expanding in recent years (Fig 2). A total of 10 large clusters with 13 to 52 Slovenian sequences and aLRT > 0.95 were observed, among which five included only sequences sampled in Slovenia (Table 4). In addition, 22 transmission pairs, two trios, and one quartet of Slovenian sequences were observed. Large clusters with at least 90% of Slovenian sequences can be seen highlighted yellow, and the remaining three clusters with a larger proportion of foreign sequences highlighted green in Fig 2. Clusters of foreign sequences can be seen nested within two of the three mixed clusters—specifically a cluster of mainly Czech and German sequences and a cluster of sequences from Serbia, indicating that HIV spread from Slovenia abroad. The sequences obtained from 2000 to 2010 and from 2011 to 2016 are mixed in the clusters, showing that the clusters are expanding. One cluster among 13 Slovenian sequences contained only sequences sampled from 2011 to 2016 and was probably only recently introduced into Slovenia.

**Table 4:** Large phylogenetic clusters (*n* > 10) of Slovenian sequences subtyped B or B-like.

Cluster	No. Slovenian sequences	(%)	Total no.	Approximate likelihood ratio test (aLRT)
1	52	98	53	0.998
2	28	90	31	0.989
3	25	100	25	0.996
4	21	70	30	0.989
5	20	100	20	0.997
6	19	53	36	0.955
7	19	100	19	0.997
8	17	35	49	0.962
9	16	100	16	1
10	13	100	13	0.978

Ten Slovenian sequences carrying SDRMs were included in the subtype B phylogenetic analysis: four sequences from this study and an additional six sequences obtained from patients diagnosed from 2000 to 2010. No forward transmission of SDRMs was observed among Slovenian sequences; in addition, only three sequences were observed to have a transmission link to other Slovenian sequences. The first sequence is part of the largest Slovenian cluster (*n* = 53) and carries the T69D mutation, the second sequence is within a cluster of 20 Slovenian sequences and carries M46I, and the third is in a phylogenetic relationship with two other Slovenian sequences and is carries K103N (Fig 2). In addition, most closely related sequences from other countries were checked for the presence of SDRMs. Only three from among seven sequences were found with a transmission link to at least one foreign sequence carrying SDRMs; all three were T215 revertant mutations. Specifically, the first Slovenian sequence, with the T215D mutation, was found most closely related to a set of sequences carrying T215C obtained from a US citizen. The second Slovenian sequence, with T215V, was nested within six sequences from the UK and Poland, all with identified T215 revertants (T215V, T215E or T215D). The third sequence, with T215S, was identified in a phylogenetic relationship with two closely related sequences from Germany, and both had the same T215S revertant mutation. A Slovenian cluster was identified that contributed to the spread of HIV in Serbia, and one person carrying Y181C, which originated from Serbia, was within a Serbian part of the identified cluster; however, none of the Serbian sequences showed the same mutational profile.

All of the phylogenetic clusters remained significant after the removal of 43 SDRM codons from the alignment, except for one cluster. Cluster 8, encompassing 49 sequences, was broken down into smaller clusters and dispersed throughout the phylogenetic tree. This cluster included the Slovenian sequence with T215V nested within foreign sequences with 215 revertants; however, the phylogenetic link remained regardless of the exclusion of SDRM sites.

Phylogenetic analysis of subtype A sequences reveled only one quartet, one trio, and one transmission pair of Slovenian sequences with aLRT > 0.95. Several individual introductions of subtype A into the country were observed (S1 Fig).

## Discussion

A low prevalence of transmitted drug resistance was determined in Slovenia for 2011–2016, and drug susceptibility was impaired the most for NNRTIs. SDRMs were found in four individuals, all MSM and infected with subtype B; however, no forward spread of TDR was observed within the country.

This study is quite representative for all of Slovenia because the random selection included 55% of individuals diagnosed with HIV from 2011 to 2016. The prevalence of TDR was estimated at 2.4% in this study period, which was half that reported for 2005–2010 (4.7%) [9]. SDRMs were detected exclusively among MSM; however, this association is not significant, possibly due to small numbers. Previous studies reported a higher prevalence of TDR among MSM compared to injecting drug users (IDUs) and heterosexuals. The odds of TDR were estimated to be higher in MSM compared to heterosexuals, and were estimated to be 28% and 42% higher in high-income and low-income countries, respectively. However, a potential bias was observed that could explain the higher prevalence of TDR because of a higher proportion of recent infections among MSM [3]. One limitation of this study is that not only recently infected individuals were selected, which possibly favored the selection of HIV variants carrying SDRMs that are equally fit to transmit as wild-type variants are. HIV-positive patients were sampled soon after the HIV-1 diagnosis; however, the HIV transmission could had taken place many years earlier, giving time for SDRMs to revert to a fitter wild type. This could have been circumvented if a more sensitive sequencing method had been employed in this study, such as next-generation sequencing or allele-specific PCR, instead of Sanger sequencing, and it is therefore possible that all existing SDRMs were not detected.

SDRMs were determined exclusively among patients infected with subtype B virus in this study, even though a higher proportion of non-B isolates was determined for 2011–2016 (17%) compared to figures reported previously (11% for 2005–2010) [9]. A study by Hofstra et al. also found a higher prevalence of TDR among subtype B isolates on account of NRTI mutations. The authors propose that this is not due to specific characteristics of subtype B strains, but is most likely a result of prolonged exposure of subtype B to NRTIs and suboptimal NRTI-monotherapy before combination ART became available [4].

Even though SDRMs were detected in equal frequency as NRTIs and NNRTIs in this study, the impact of mutations on drug susceptibility was most pronounced for the NNRTIs, as previously observed [4]. Less high-level resistance was observed in 2011–2016 compared to 2000–2010, especially in the case of currently recommended treatment combinations. This indicates that no modification is needed in the national strategy for routine pre-treatment drug-resistance testing; currently only pregnant women and individuals most likely infected abroad are tested. In addition, the GSSs obtained indicate that no PI-resistant strains are circulating in Slovenia, potentially allowing simplification of treatment for selected patients by means of ritonavir-boosted PI monotherapy (PI/r) with darunavir or lopinavir, or a dual therapy with lamivudine added to PI/r.

Forward spread of HIV variants carrying SDRMs has not yet been observed in Slovenia, with not even a single transmission pair identified. Thus it seems that TDR in Slovenia either results from separate introduction events of the virus into the country or results directly from unsuccessfully treated individuals. Only three Slovenian sequences with SDRMs were observed nested within the identified Slovenian clusters and could potentially be contributing to forward transmission of SDRMs in the country, yet none have been observed, possibly due to decreased transmission fitness of these mutations. Indeed, the study by Wertheim et al. (2017) observed significantly less clustering of mutations T69D and M46I detected in the two sequences within large Slovenian clusters, which indicates that these two mutations are less fit to transmit than the wild type [22]. Among the other seven Slovenian SDRM sequences with a phylogenetic link to foreign sequences, only three sequences with T215 revertant mutation were found to have a transmission link to a sequence sampled abroad and exhibiting the same SDRMs. The T215 revertants appear in the absence of drug pressure from T215Y/F mutations that were initially selected under zidovudine treatment. In contrast to T215Y/F, T215 revertants show no reduction in transmission fitness [22,23]. Equally fit are K103N, K103S, and Y181C mutations [22], which were detected in four individuals from Slovenia. However, no phylogenetic links to other foreign sequences carrying these SDRMs were observed, which could be explained by the lack of sampling or because these SDRM strains had just recently been transmitted from treatment-experienced patients.

Subtype B was determined in the majority of individuals (83%), and an even higher proportion was observed among MSM (87%). Previous analysis of the dynamics of the subtype B epidemic in Slovenia, performed on a dataset obtained from patients diagnosed from 2000 to 2012, revealed eight large Slovenian clusters with ≥ 10 sequences and less than 20% of sequences without a transmission link to another Slovenian sequence. Only one pair of foreign sequences was nested within one large Slovenian cluster; otherwise, clusters encompassed only Slovenian sequences [24]. In contrast, this analysis included additional sequences from 2013 to 2016 and showed more intermixing of nationalities within large clusters because half of large clusters harbored sequences sampled abroad. Moreover, the forward spread of HIV was observed from Slovenia to Serbia, and to Germany and the Czech Republic. This finding is in line with data from an observational study of the sexual behavior of MSM in Slovenia that showed that individuals diagnosed with HIV after 2014 more often reported they most likely acquired HIV infection abroad and exhibited more high-risk sexual practices overall (e.g., the use of mobile apps or websites to search for sex partners, attending MSM parties, and use of recreational drugs during sex, or “chemsex”) in comparison to individuals diagnosed from 2009 to 2013 [25]. This is indirectly supported by a greater number of syphilis cases found among this study population (21%) compared to 2005–2010 (17%), for the most part among MSM (94%). In addition, this study suggests that all large Slovenian clusters are expanding and an additional cluster of solely Slovenian sequences was detected, possibly introduced to Slovenia in the last 5 years. Furthermore, this cluster included 7/13 patients with a documented syphilis infection. Overall more syphilis cases were observed among individuals carrying subtype B compared to those infected with non-B subtypes, again likely on account of riskier behavior of MSM because subtype B was found to be significantly associated with this transmission route.

Two-thirds of individuals infected with a non-B subtype reported a heterosexual mode of HIV transmission in comparison to only 11% of patients with subtype B virus. Moreover, non-B subtypes were determined significantly more often among women and immigrants. This was previously observed in a Europe-wide study examining the characteristics of HIV subtypes circulating in Europe. Subtype B was found to be most prevalent and determined significantly more often among MSM compared to IDUs and heterosexuals; however, the authors anticipated an influx of different subtypes into Europe in the near future [26]. Dispersal of non-B subtypes within a local (native) population was already observed in various central and western European countries; for example, subtype G in Portugal, subtype A in Greece, and subtype C in the UK [27–33]. Consistent with these findings are more non-B infections observed among MSM in 2011–2016 as opposed to 2005–2010; namely, 6.2% vs. 2.5% [9].

In conclusion, the prevalence of TDR was estimated at 2.4% among individuals diagnosed with HIV-1 in 2011–2016 in Slovenia. Forward spread of TDR has not been observed in the country thus far; however, phylogenetic analysis revealed several new introductions of HIV to Slovenia, possibly due to increased risky behavior by MSM noted in recent years. This was indirectly confirmed by a substantial increase in syphilis cases and HIV-1 non-B subtypes during the study period, suggesting that the introduction of a drug-resistant HIV variant with good transmission fitness is a likely event in the near future, and emphasizing the importance of continuing and close surveillance of TDR in Slovenia.

## Sequence data

The GenBank accession numbers of the sequences included are:

KF753699-KF753751, KP013639-KP013667, KY656612-KY656628, KY656630-KY656636, KY656638, KY656640-KY656649, KY656651-KY656667, KY656669-KY656674, MF987697-MF987724.

## Supporting information

S1 Fig Maximum likelihood phylogenetic tree of Slovenian sequences subtyped A. Slovenian sequences from the 2011–2016 dataset are colored green, sequences from the 2000–2010 dataset are colored blue, and corresponding control sequences are shown in black.
(TIFF)
