# Supplementary figures and images for: HIV-1 transmitted drug resistance in Slovenia and its impact on predicted treatment effectiveness: 2011–2016 update
Source: PLoS One. 2018 Apr 26;13(4):e0196670. doi: 10.1371/journal.pone.0196670 (PMC5919638; doi:10.1371/journal.pone.0196670)

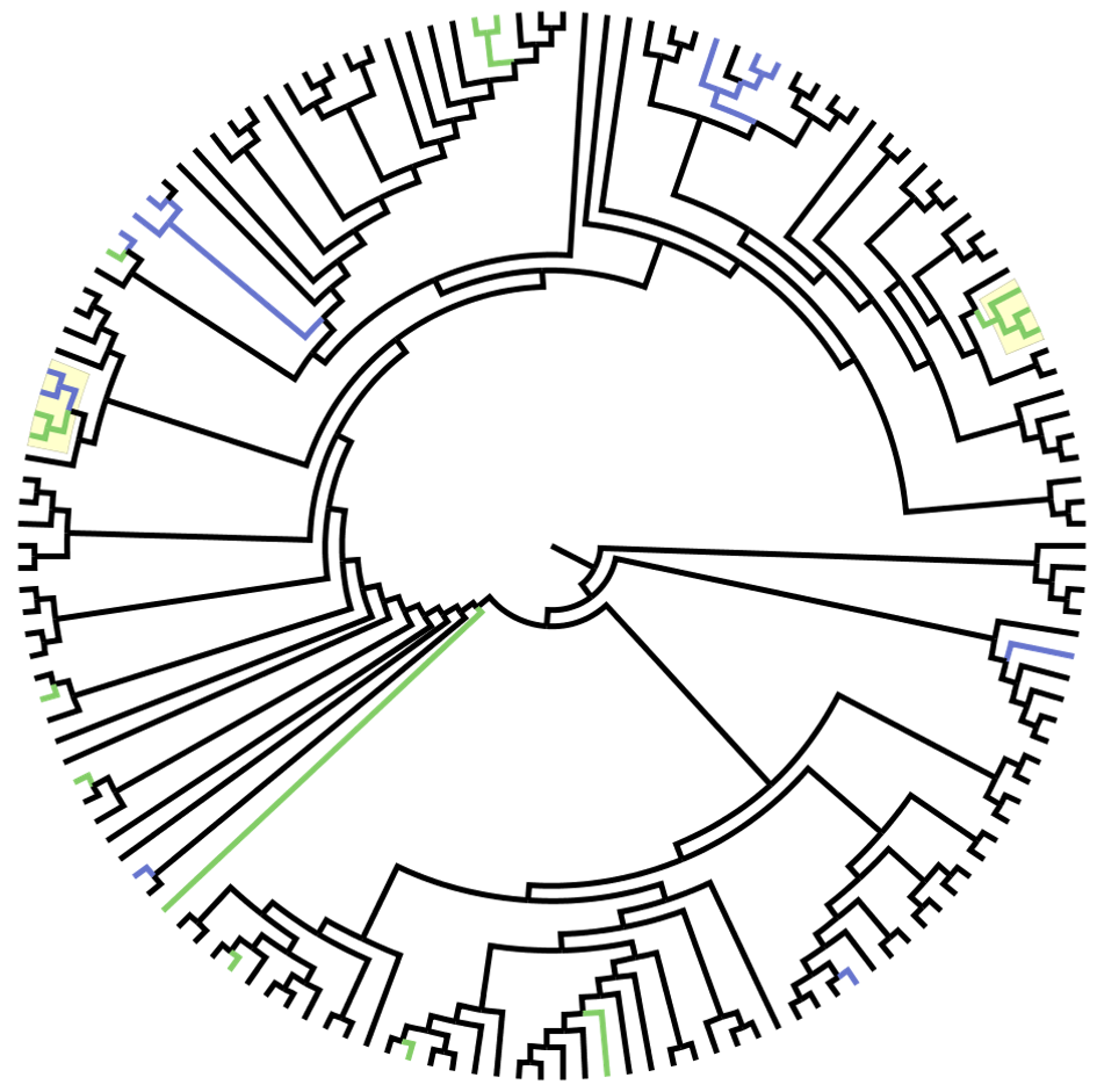

Supplement: S1 Fig — Slovenian sequences from the 2011–2016 dataset are colored green, sequences from the 2000–2010 dataset are colored blue, and corresponding control sequences are shown in black. (TIFF) [file pone.0196670.s001.tiff]
